# Detection of In-Plane Movement in Electrically Actuated Microelectromechanical Systems Using a Scanning Electron Microscope

**DOI:** 10.3390/mi14030698

**Published:** 2023-03-22

**Authors:** Tarmo Nieminen, Nikhilendu Tiwary, Glenn Ross, Mervi Paulasto-Kröckel

**Affiliations:** Department of Electrical Engineering and Automation, Aalto University, Otakaari 5, 02150 Espoo, Finland

**Keywords:** scanning electron microscope (SEM), motion detection, MEMS, in-plane motion

## Abstract

The measurement of in-plane motion in microelectromechanical systems (MEMS) is a challenge for existing measurement techniques due to the small size of the moving devices and the low amplitude of motion. This paper studied the possibility of using images obtained using a scanning electron microscope (SEM) together with existing motion detection algorithms to characterize the motion of MEMS. SEM imaging has previously been used to detect motion in MEMS device. However, the differences in how SEM imaging and optical imaging capture motion, together with possible interference caused by electrical actuation, create doubts about how accurately motion could be detected in a SEM. In this work, it is shown that existing motion detection algorithms can be used to detect movement with an amplitude of 69 nm. In addition, the properties of SEM images, such as bright edges, complement these algorithms. Electrical actuation was found to cause error in the measurement, however, the error was limited to regions that were electrically connected to the actuating probes and minimal error could be detected in regions that were electrically insulated from the probes. These results show that an SEM is a powerful tool for characterizing low amplitude motion and electrical contacts in MEMS and allow for the detection of motion under 100 nm in amplitude.

## 1. Introduction

Microelectromechanical systems (MEMS) were adopted to create several different devices from microphones [1] and inertial sensors [2,3] to energy harvesters [4] and micromirrors [5,6]. These devices display different motions, and with devices becoming increasingly complex and miniaturized, more advanced characterization methods have sought to accurately analyze their motion.

Traditionally, the motion of MEMS has been limited to in-plane motion and out-of-plane motion. Motion is an important characteristic of MEMS and can provide information about important properties, such as the piezoelectric coefficient [7,8], or of the general functioning of the device [9,10,11]. Hence, the availability of methods for detecting this motion is important. The high-accuracy detection of out-of-plane motion in MEMS has been performed using laser Doppler vibrometry [12] and various interferometric methods [9,13,14,15,16]. However, these methods are not able to characterize in-plane motion, since the motion must be in the direction of the incident beam. Efforts have been made to perform these measurements by tilting the moving sample to partially align the motion with the laser [17], positioning the laser at a low angle [9] and fabricating an optical fiber next to the MEMS to direct the laser parallel to the motion [13], or by optical knife-edge measurement [18]. However, these are complicated setups and place requirements in terms of the design of the device or require several extra fabrication steps simply to enable their characterization. The best-performing method for characterizing in-plane motion in MEMS is that of digital holographic microscopy (DHM) [19,20], which can be used to measure in-plane and out-of-plane movement in MEMS up to 25 MHz and has been used to detect movement at the nanometer scale [21]. However, DHM might not be available at all locations, and in many cases, it is not feasible to spend resources on a device for a single measurement, especially if the motion of MEMS is not regularly measured. Thus, an easy and inexpensive method for characterizing MEMS motion would be highly beneficial.

Even though the in-plane motion is difficult to accurately characterize, it can be visually detected using a microscope. This lead to the creation of several algorithms to calculate the amplitude of motion using an optical microscope [10,22,23,24]. These algorithms have demonstrated a high-accuracy characterization of in-plane motion in MEMS. However, they share three main challenges hurdling the detection of movement in MEMS: capturing low-amplitude motion, capturing high-frequency motion, and achieving a high contrast between the device and the substrate. The first problem is obvious, as movement in MEMS is in the micrometer—or even nanometer—range, which means that high-resolution imaging is required in order to even detect the motion. Modern optical microscopes are able to detect motion in the range of hundreds of nanometers, after which the optical resolution limit is reached [25]. The second problem relates to the high-frequency motion of MEMS. Motion in the range of kHz or MHz frequency is difficult to capture without including a large amount of motion blur in the image. This is especially problematic for algorithms, such as that presented in [23], where images of a non-moving device are compared to calculate the motion. The high-frequency motion can be captured by means of a stroboscopic setup [15], which consist of short light pulses that are synchronized to the motion of the device. The light sources create the only illumination for the sample, effectively ignoring the exposure time of the camera. The stroboscopic approach does not inherently have limitations to the amplitude of motion it can detect, as it does not detected the motion in itself, but has to be paired with another method to detect the motion. Another solution is to utilize motion blur [22,24] to characterize the motion in which case the high-frequency motion poses no problems for the algorithm. Finally, a problem that has to be solved is distinguishing the moving device from the background. Low contrast between the moving device and the background makes it challenging to separate device movement from the noise, reducing the accuracy of the measurement.

These problems could be addressed by means of a scanning electron microscope (SEM) instead of an optical microscope. Due to the superior resolution of SEMs, the detection of movement is possible all the way to the nanometer range. Another beneficial property of SEM imaging is that the edges of the sample appear brighter in the image thanks to the larger surface area emitting electrons (known as the edge effect), which allows for the easy detection of moving surfaces and provides a strong contrast between the substrate and the moving edge. This also benefits algorithms that fit a function to the intensity data by providing an easily distinguishable, moving feature for the fitting. SEM imaging has already been used to perform the rudimentary analysis of MEMS motion [26,27,28]. However, several open questions remain about the possibility of utilizing SEM images as input for motion detection algorithms to accurately characterize in-plane motion, such as compatibility with electrical actuation.

This paper aimed to answer these questions and demonstrate SEM imaging as a tool to characterize in-plane motion in MEMS. To achieve this aim, the study investigates and analyzes two electrically actuated MEMS, a capacitive comb-drive and a piezoactuated microcantilever.

## 2. Materials and Methods

**Figure 1:**
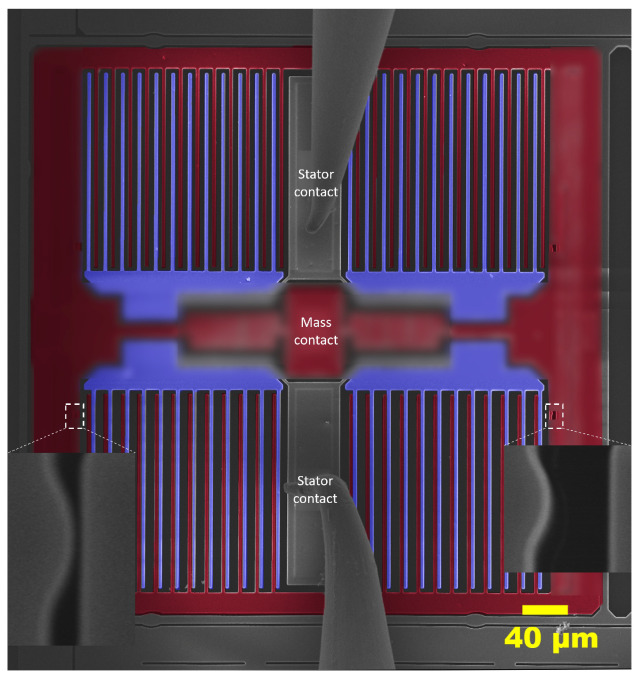
Structure of the comb-drive used in the measurements. The red area denotes the moving mass, which is used for calculating the motion. The blue area denotes stationary fingers, which are used as a reference. Motion limiters are highlighted as the known gap between the moving structure and the bump is used as the reference when confirming the results.

**Figure 2:**
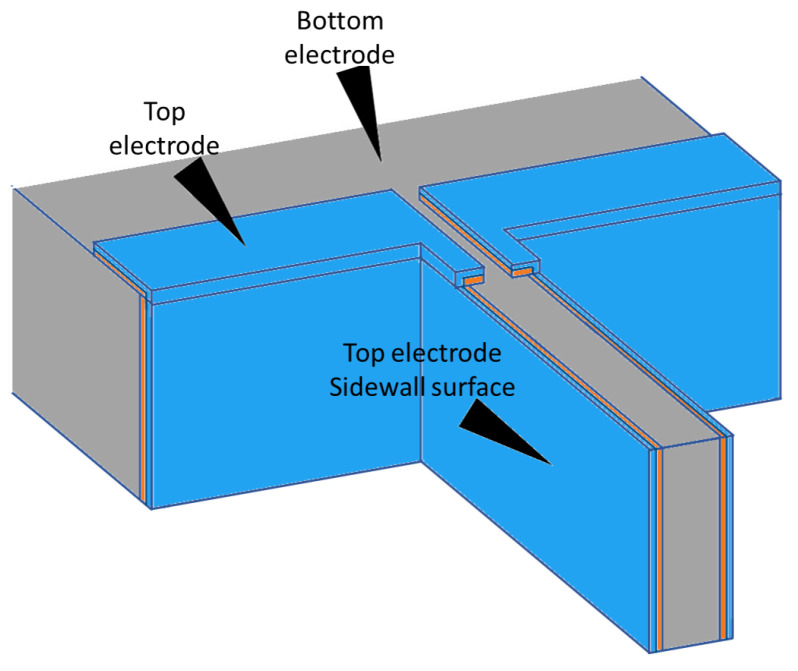
Structure of the microcantilever used in the measurements. The microcantilever consists of a 300 nm AlN (represented in the image as an orange layer) and a 100 nm TiN thin films (represented in the image as a blue layer) located on the sidewalls and on the top of the back wall. Conductive silicon is used as a bottom electrode. The sample was probed at three locations: bottom electrode, top electrode, and sidewall electrode. These locations are shown in the image.

Two different samples were used for the experiments: a capacitive comb-drive sensor provided by Murata Electronics Oy and a piezoelectrically actuated microcantilever. The structure of the comb-drive is presented in Figure 1. Since the comb-drive is used to only generate motion to be detected, its application and dimensions are not important, with the exception of the motion limiters, which are highlighted in Figure 1. The motion limiter consists of a stationary finger and a moving finger. The stationary finger has a small bump that the moving finger collides with, stopping the motion. The gap between the bump of the stationary finger and the moving finger at rest position determines the amplitude of the motion and is referred to as the limiting gap. The microcantilever consists of a 300 nm thick aluminum nitride (AlN) layer as the piezoelectric material and 100 nm thick titanium nitride (TiN) layer as the top electrode, with an n-type silicon acting as the bottom electrode. Both of the thin films were deposited on the vertical surfaces of the cantilever and on top of a back wall as probing pads, as shown in Figure 2.

**Figure 3:**
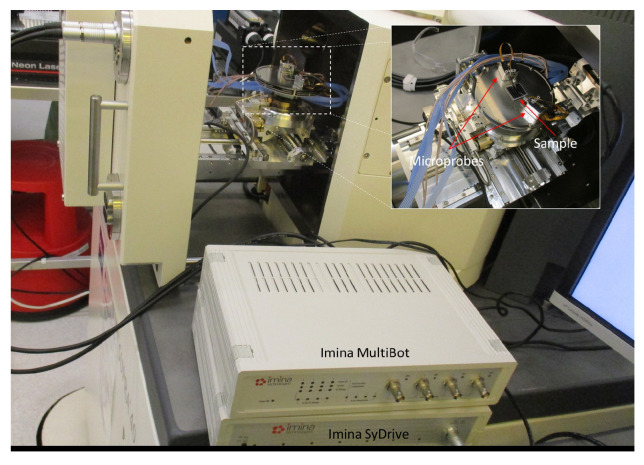
Experimental setup used in the characterization of the samples. The sample is actuated with two Imina nanoprobes that are connected to Imina MultiDrive, Imina syDrive, and Rigol 1022 (not pictured) using feedthrough cables.

The characterization was performed using a Zeiss Supra 40 SEM and the signal for electrical probing was created using a Rigol DG1022, which was connected to the SEM using an Imina nanoprobe setup. The measurement setup is shown in Figure 3. The comb-drive samples were characterized using a 1 Hz square wave with a 0 V${}_{DC}$ bias and a 11 V${}_{pp}$ amplitude and the images used in the motion analysis were captured using the fastest scanning speed on the SEM, with a scan time of 122.3 ms and a speed of 0.15 μs/px. The comb-drives were actuated using a 1 Hz square wave to demonstrate the possibility of using the method on samples that move during imaging, however, the frequency was kept small to preserve the DC-like actuation. The microcantilevers failed to demonstrate motion during the experiments but were used to determine the impact of electrical actuation as they had more suitable electrodes than the comb-drives. As such, they were characterized using a 1 Hz sine wave with a 0 V${}_{DC}$ bias and a 20 V${}_{pp}$ amplitude and the images were captured using a slower scanning speed, with a scan time of 20.2 s and a speed of 25.1 μs/px. In addition, the observed electrical effects in the SEM were confirmed by performing an I-V sweep using a Keysight B1500A Semiconductor Device Analyzer. Both InLens (which uses secondary electrons from the surface of the sample) and SE2 (which uses secondary electrons from below the surface of the sample) detectors were used to capture the images.

In order to determine the motion from the SEM images, an algorithm demonstrated by Kokorian et al. [23] was used. This algorithm was chosen as it was shown to be able to detect very small motions and the authors believed it would complement the features of SEM images. The working principle of the algorithm is described below, however, for a complete understanding of the algorithm, the authors recommend referring to the original work. To determine the amplitude of displacement, the algorithm requires two images of the moving device: an image of the sample before it has moved, and an image of the sample after moving. A spline interpolation function is used to create an algebraic representation of the intensity profiles of these two images. These splines can be displaced according to a function $f\left(x\right)=A\cdot s\left(x-x_{0}\right)+y_{0}$, where $x_{0}$ is the shifted parameter, s(x) is the spline interpolation function, *A* and $y_{0}$ describe the amplitude and offset, respectively. When the spline of the device before motion has been displaced on top of the second spline, the value of $x_{0}$ describes the amplitude of displacement in pixels. To reduce the effect of noise on the algorithm, the images were compressed to a single row by averaging the values in a column and using the resulting row as the intensity profile for fitting the spline. In this work, the spline was displaced in increments of 0.01 px and a mean squared error (MSE) between the two splines was calculated. The displacement with the lowest MSE was determined to be the actual amplitude of deflection. To reduce the effect of captured noise and variations in the movement, hundred SEM images were averaged to create the images of the sample before and after displacement. This process is explained in detail in Section 3.1. The displacement was calculated from the created images using the previously mentioned algorithm. All of the analysis was performed using Python 3.9.13.

## 3. Results and Discussion

### 3.1. Capture of SEM images Containing Motion

**Figure 4:**
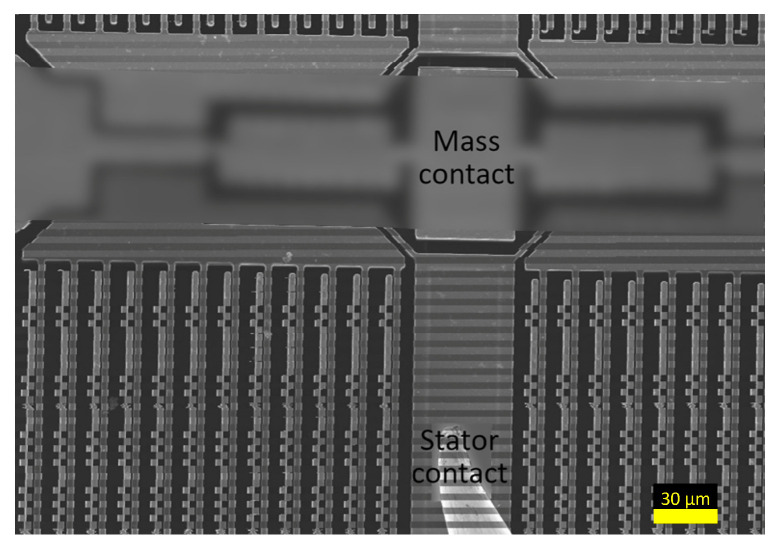
Image of the moving comb-drive taking using a scanning speed of 25.1 μs/px. The changes in intensity are caused by the electrical actuation and are discussed in more detail in Section 3.2.

When moving devices are imaged in an SEM, the motion is captured as disjointed, where the moving sample changes location as shown in Figure 4. This leads to challenges when characterizing the amplitude of the motion, since common noise reduction methods, such as averaging the image into a single row, cannot be used when the image contains the moving device at different parts of the motion. However, these challenges can be circumvented by selectively combining features from several images where the moving device is located in the same area. This can be achieved by classifying each pixel in an image into two categories: device in rest position or device in actuated state.

**Figure 5:**
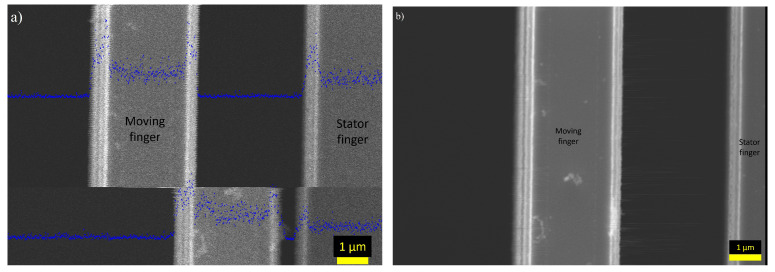
(**a**) Image of a comb-drive finger in motion. The intensities are plotted over the image in blue. The intensity peaks for the two possible positions of the finger can be clearly distinguished, making the selective combining easy. (**b**) A selectively combined image of the finger in the left location. The selective combination was performed from a set of 100 SEM images.

This classification can be performed by taking advantage of the edge effect seen in SEM images. Intensity peaks created by the edge effect are easily distinguishable from the intensity profile and can be used to determine the location of the moving device by calculating the center of the intensity peak. This effect emphasizes the extremities of the motion as the device pauses for a brief moment before switching directions, ensuring that the edge effect is captured in its entirety. The classified pixels can then be averaged into a new image showing a stationary shot of the moving sample. This is shown in Figure 5a,b. It can be observed from Figure 5b that the drift is not an issue for the formation of the images. This is evident from the fact that the focus of the image is good, even though it is a composite of 100 images takes over several minutes. An additional benefit of combining several images to represent the motion is that the variations and errors in the movement are cancelled and the average deflection can be determined.

In order to successfully combine several images, it is important that the frequency of the moving device is not too high when compared to the scanning speed of the SEM to minimize the amount of motion blur contained in each pixel. This condition is fulfilled when $f_{\mathrm{device}}\leq \frac{f_{\mathrm{SEM}}}{2\times 10}$, where $f_{\mathrm{device}}$ is the actuation frequency of the device, and $f_{\mathrm{SEM}}$ is the scanning frequency of one pixel of the SEM. The SEM frequency is divided by 2 so one pixel records only half of the full range of motion (from stationary to maximum deflection) and again by 10 so a tenth of deflection is contained in a single pixel. The SEM used in this work has a scanning speed of 0.15 μs/px, which is a frequency of 6.4 MHz, giving the maximum frequency of 320 kHz that can be detected. However, it is possible that higher frequencies could be detected using motion detection algorithms that do not require an image of the sample at the maximum deflection, such as that demonstrated by Burns and Helbig [22].

**Figure 6:**
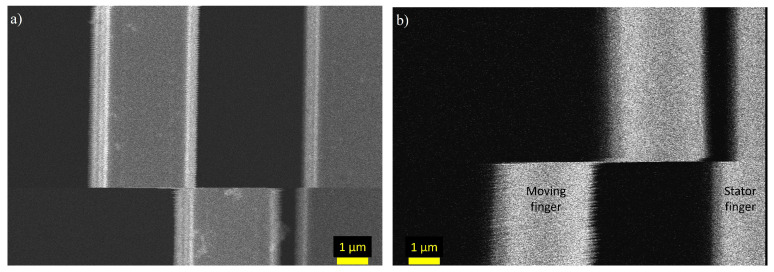
(**a**) Comb-drive motion captured using the InLens detector. From the image, it can be seen that the edges have more details and particles and defects on top of the fingers can be detected. In addition, a clear change in intensity can be seen on the stationary finger. (**b**) Comb-drive motion captured using the SE2 detector.

Finally, InLens and SE2 detectors were compared for the image capturing process, and images taken using both detectors are shown in Figure 6. Images taken using the InLens detector show features, such as particles or defects, and the edges can be very clearly seen as they have a significantly higher intensity than the background or the middle part of the comb finger. It was also observed that the electrical actuation had two effects on the image, an intensity change and a slight displacement of the structure. The small features that could be seen in the InLens images were not visible in the SE2 detectors images. The edge effect in these images was not as strong, making it more difficult to detect the edge. However, the contrast between the finger and background was higher, so detecting the finger from the background was still possible. There was also no intensity change caused by the electrical actuation, however, the change in surface bias voltage still caused the displacement of the image. To make selective combining easier, images used in further analyses were taken using the InLens detector.

### 3.2. Impact of Electrical Actuation

As mentioned in the previous section, electrical actuation has two significant effects on the image. Firstly, the brightness of the images changes based on the actuation. Secondly, a slight displacement is visible in the regions that experienced the brightness change. These effects are caused by the surface charging as a result of the electrical actuation, which in turn, affects the secondary electron yield. When a negative voltage is applied, the surface is charged positively, reducing the secondary electron yield and creating a darker image. Conversely, when a positive voltage is applied, the surface is negatively charged, increasing the secondary electron yield and the brightness of the image. This effect can be seen in Figure 4. It can also be seen that the moving fingers do not experience the charging to the same extent as the stationary fingers. This is a result of the moving fingers being electrically insulated from stationary fingers, which are directly connected to the probe. As such, they do not experience the same voltage change and are not charged.

**Figure 7:**
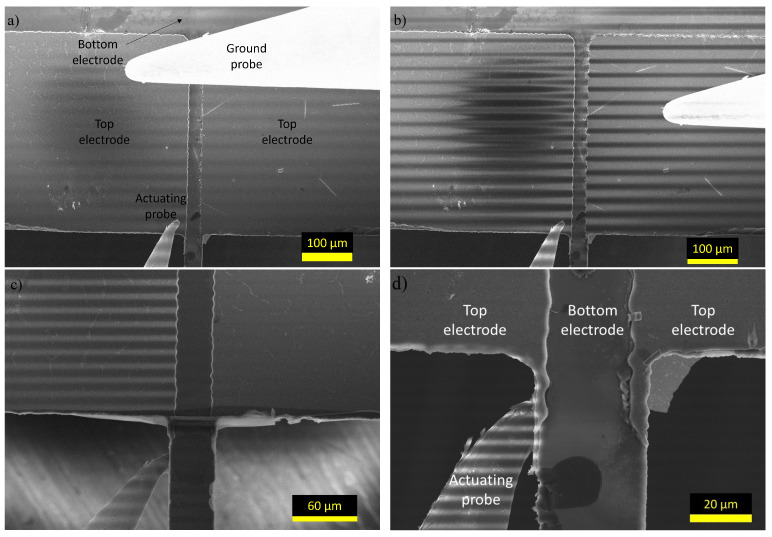
SEM images of microcantilevers under electrical actuation. (**a**) Probes on the same electrode; (**b**) Probes on different, not connected, electrodes; (**c**) Probes on a sidewall with an electrical connection to the top electrode; and on the bottom electrode (Si); and (**d**) probes on a sidewall with no electrical connection to the top electrode and on the bottom electrode. Sub-figures (**a**,**b**,**d**) are of the same cantilever.

This effect is even more pronounced for the microcantilevers, where a piezoelectric AlN layer acts as an insulating layer between the top electrode and silicon. By comparing the patterns in Figure 7a,b, it can be determined that the surface charging is the reason for the pattern and what defines the intensity of the patterns. When the probes are placed on the same electrode, the surface does not charge as much as when they are on different electrodes, leading to a less pronounced pattern. In addition, it can be seen that the pattern is the same phase for the entire image in Figure 7a. When the probes are on opposing electrodes, which are insulated from each other by an AlN layer, a voltage difference is created between the two electrodes, creating a very strong pattern in Figure 7b. It can also be seen that the pattern on the two electrodes are out-of-phase, showing that when the left electrode has a positive potential, the right electrode has a negative potential and vice versa. Figure 7c,d demonstrate how a broken electrical connection affects the pattern. In Figure 7c, an electrical connection between the electrode on the sidewall and the electrode on the top exists, meaning that both electrodes experience the surface charging caused by the actuation. Opposed to this, in Figure 7d, there is no connection between the sidewall and top electrodes meaning that probing the sidewall does not affect the charge of the top electrode. This was also confirmed through I-V measurements between the sidewall electrode and top electrode. The I-V curve measured for the connection shown in Figure 7c was linear and the measured current was in the mA scale. Conversely, the connection from Figure 7d was not linear and had a current in the μA scale.

This suggests that, if the moving structure is electrically insulated from the electrodes, it should experience minimal noise from the surface charging. In addition, the surface charging is dependent on the magnification. When the magnification is low, the amplitude of the noise created by surface charging is large, and when the magnification is high, the amplitude of the noise is small. Therefore, the effect of the noise can be reduced by increasing the amplitude and increasing the deflection-to-noise ratio.

### 3.3. Calculated Deflection

**Figure 8:**
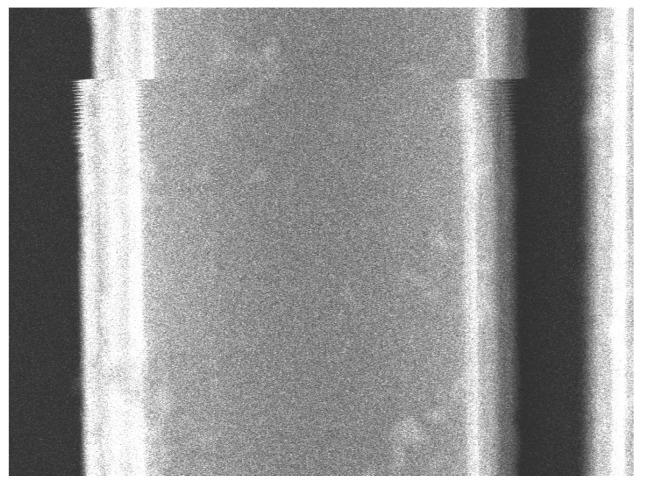
Motion from mechanical noise captured in the SEM imaged using a magnification of 75 kx.

To accurately calculate the displacement of the comb-drive, two types of motion were detected: deflection during actuation and deflection caused by mechanical noise, which is shown in Figure 8. Two images, containing the deflected and non-deflected state of the device for both types of deflection, were created by selectively combining 100 images. The deflection was calculated from these images using the algorithm developed by Kokorian et al. [23].

**Figure 9:**
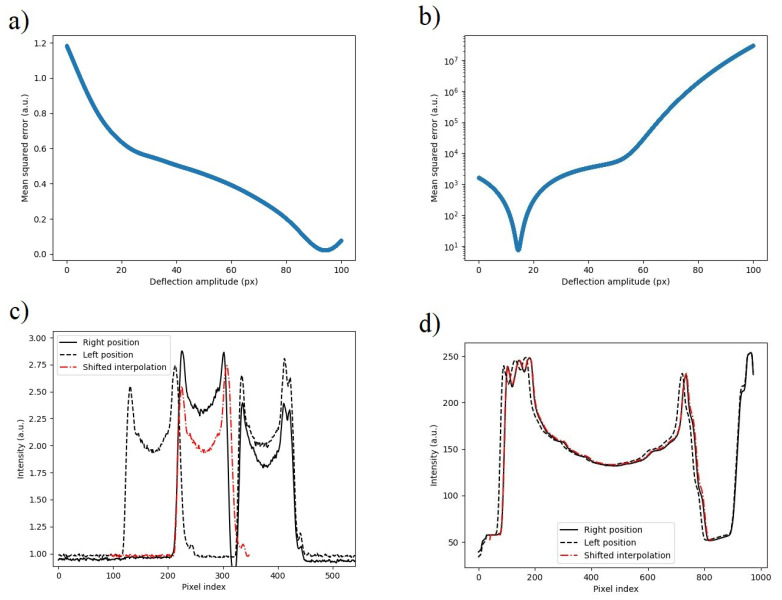
(**a**) Mean squared error (MSE) for the curve-fitting process of the actuated displacement. (**b**) Mean squared error from the curve-fitting process for the mechanical noise. (**c**) Illustration of the spline at the displacement value with lowest error for actuated displacement. (**d**) Spline displacement for mechanical noise. The large difference in the scale of (**a**,**b**) is the result of the intensity difference between (**c**,**d**), which is amplified when taking the MSE. However, for the functioning of this algorithm the maximum intensity is not as important as the ratio of background intensity and intensity of the moving device.

**Figure 10:**
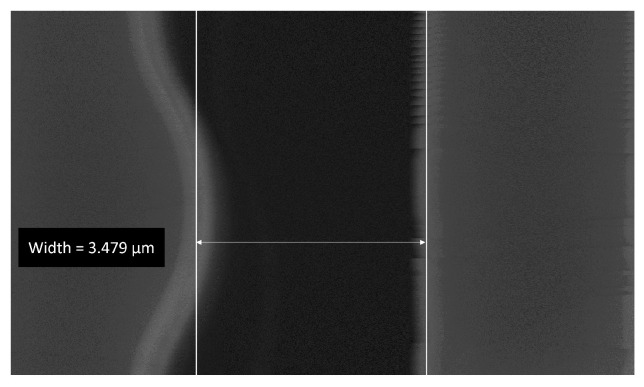
Limiting gap measured using a tool built-in the SEM software.

The calculated MSEs are shown in Figure 9a,b for actuated deflection and deflection from mechanical noise, respectively. The smallest errors were achieved with the displacements of 93.92 pixels for actuated deflection and 14.41 px for mechanical noise. Using the spatial resolutions, 35.71 nm/px actuated deflection and 4.76 nm/px for noise, the total displacement of the comb-drive was calculated to be 3354 nm ± 69 nm. The intensity plots showing the displaced spline with the minimum error alongside the original intensities is presented in Figure 9c,d for actuated deflection and noise, respectively. These results are well matched with the limiting gap of the comb-drive, which was measured to be 3479 nm. The SEM image of the limiting gap is shown in Figure 10. The difference of roughly 100 nm is likely the result of the manual placement of measurement lines.

## 4. Conclusions

This work explored the possibility of utilizing an SEM to characterize motion in electrically actuated MEMS. We determined that small amplitude motion, which is impossible to capture using a traditional microscope, can be detected using known motion detection algorithms in combination with SEM imaging. The method whereby SEM captures the images further benefits its use for detecting motion as it allows for the capture of motion at high frequencies—up to 320 kHz for the SEM used in the study. Higher-frequency motion has been detected using SEM [28], however, such levels of motion likely require different motion detection algorithms for an accurate estimation due to the significant amount of motion blur. This is significantly higher than what is possible using a standard optical microscope without any additional features. In addition, the edge effect seen in the SEM images benefits most motion detection algorithms, allowing them to clearly distinguish the moving sample from the substrate, and giving them a clear focal point to use for determining the movement. The edge effect also allows an easy way to selectively combine images to visualize the stationary position from several images containing motion.

The motion of two different amplitudes were characterized in this work: 3354 nm actuated deflection and 69 nm mechanical noise. Motion with an amplitude of 3354 nm can be easily characterized using existing optical methods to obtain high accuracy. However, motion with an amplitude of 69 nm cannot be detected using existing optical methods as the motion would be contained in a single pixel and thus could not be measured accurately. Electrical probing was found to add sources of error in the measurement, which are not present in the optical imaging. The change in surface charge caused by the electrical actuation created noticeable motion in the image and created a change in focus between the images that were actuated with a different voltage. These effects were limited to regions that had an electrical connection with the area being probed and did not affect the motion detection if the moving sample had a region that was not in electrical contact with the probe, and had some focal point that could be tracked. The impact of this noise should be characterized in depth in future work to understand the accuracy when characterizing small amplitude motion. In addition, since the effects were only seen on regions with an electrical connection to the probe, it is possible to utilize SEM to visualize an electrical connection and to observe short circuits or leaks in the system.

In summary, SEM imaging shows great potential as an easy method for detecting low-amplitude motion in electrically actuated MEMS. Whilst a magnification of 75 kx below 100 nm could be detected, suggesting that higher magnifications could be used to image even smaller movements, however, this must be confirmed with further research as an increase in magnification can lead to other issues, such as drift and poor focus. Based on the results of this study, using SEM to detect the motion offers higher precision than an optical microscope but has a lower precision and frequency limit than DHM. However, even with the limitations in the frequency, the method demonstrated in this publication would be sufficient to characterize the majority of in-plane displaced MEMS devices using a tool that is commonly available in research facilities, and thus providing a good alternative solution for characterizing in-plane motion. As a result, SEM imaging is a potent method for characterizing MEMS with complicated motion. However, further research should be conducted to characterize high-frequency devices and devices with movement in the range of tens of nanometers to determine the limitations of the approach.

## Data Availability

Data used in the study are available upon request from the corresponding author.

## Abbreviations

The following abbreviations are used in this manuscript:

MEMS	Microelectromechanical systems
DHM	Digital holographic microscopy
AlN	Aluminum nitride
TiN	Titanium nitride
MSE	Mean squared error
